# *Salmonella* Type III Secretion Effector SrfJ: A Glucosylceramidase Affecting the Lipidome and the Transcriptome of Mammalian Host Cells

**DOI:** 10.3390/ijms24098403

**Published:** 2023-05-07

**Authors:** Julia Aguilera-Herce, Concepción Panadero-Medianero, María Antonia Sánchez-Romero, Roberto Balbontín, Joaquín Bernal-Bayard, Francisco Ramos-Morales

**Affiliations:** 1 Departamento de Genética, Facultad de Biología, Universidad de Sevilla, Avda Reina Mercedes, 6, 41012 Sevilla, Spain; jaguilera3@us.es (J.A.-H.); conchipanaderomedianero@gmail.com (C.P.-M.); rbalbontin@us.es (R.B.); jbbayard@us.es (J.B.-B.); 2 Departamento de Microbiología y Parasitología, Facultad de Farmacia, Universidad de Sevilla, Avda Reina Mercedes, 6, 41012 Sevilla, Spain; mtsanchez@us.es

**Keywords:** *Salmonella enterica*, type III secretion systems, SrfJ, ceramide, CCL5

## Abstract

Type III secretion systems are found in many Gram-negative pathogens and symbionts of animals and plants. *Salmonella enterica* has two type III secretion systems associated with virulence, one involved in the invasion of host cells and another involved in maintaining an appropriate intracellular niche. SrfJ is an effector of the second type III secretion system. In this study, we explored the biochemical function of SrfJ and the consequences for mammalian host cells of the expression of this *S. enterica* effector. Our experiments suggest that SrfJ is a glucosylceramidase that alters the lipidome and the transcriptome of host cells, both when expressed alone in epithelial cells and when translocated into macrophages in the context of *Salmonella* infection. We were able to identify seventeen lipids with higher levels and six lipids with lower levels in the presence of SrfJ. Analysis of the forty-five genes, the expression of which is significantly altered by SrfJ with a fold-change threshold of two, suggests that this effector may be involved in protecting *Salmonella* from host immune defenses.

## 1. Introduction

Type III secretion systems (T3SS) are found in many Gram-negative pathogens and symbionts of animals and plants, including members of the genera *Salmonella, Shigella, Yersinia, Rhizobium, Escherichia,* and *Pseudomonas* [1]. These systems, evolutionarily related to bacterial flagella, are similar to molecular needles that span the inner membrane, periplasmic space, outer membrane, and host cell membrane to translocate proteins, called effectors, into the cytosol of the host cell cytoplasm [2]. Bacteria of the species *Salmonella enterica* infect humans and other animals causing a variety of diseases, including gastroenteritis, abortion, and typhoid fever, depending on the serotype of *S. enterica* and the host involved. These bacteria possess two distinct T3SSs, encoded in *Salmonella* Pathogenicity Islands (SPIs), which secrete about 40 effectors [3,4]. SPI1-encoded T3SS (T3SS1) is important for the invasion of host cells [5]. Once inside the cell, *Salmonella* survives and replicates in a modified phagosome known as the *Salmonella*-containing vacuole (SCV) [6]. The SPI2-encoded T3SS (T3SS2) is expressed in response to environmental cues that are found in the SCV. Effectors secreted by this system are essential to maintain SCV and to allow *Salmonella* survival within phagocytic cells [7]. These T3SS have been mainly studied in *S. enterica* serovar Typhimurium. This serovar, which causes gastroenteritis in humans, produces a systemic disease in susceptible mice that is similar to typhoid fever caused in humans by the serovars Typhi and Paratyphi. Mice pretreated with antibiotics develop acute intestinal inflammation in response to oral infection with *S. enterica* serovar Typhimurium, providing a model for the study of *Salmonella*-related gastrointestinal diseases [8]. Experiments carried out in this host have indicated that T3SS1 is important for gastrointestinal diseases, but dispensable for systemic infection [9], while T3SS2 is necessary for both types of infections [10,11].

The T3SS2 delivers about 30 effectors from intracellular bacteria across the SCV membrane. Only two of these effectors, SseF and SseG, are encoded in SPI2 [12]. The rest are encoded outside the island and, like SPI2 itself, were also acquired by horizontal gene transfer. Given the variation in the repertoire of T3SS2 effectors among different strains and serovars, these effectors can be classified into three groups [7]: core effectors (PipB, PipB2, SifA, SseF, SseG, SteA, and SteD), effectors present in intestinal serovars (SifB, SlrP, SopD2, SseJ, SseK2, SseL, SteB, and SteC), and accessory effectors (GogA, GogB, GtgA, GtgE, SpvB, SpvC, SpvD, SrfJ, SseI, SseK1, SseK3, SspH1, SspH2, and SteE). The effectors of T3SS2 are involved in tethering SCVs to the Golgi network, the production of *Salmonella*-induced tubules, interference with lysosome function and trafficking, the remodeling of actin cytoskeleton, and the manipulation of innate immunity [7]. Most effectors have diverse enzymatic activities and/or functions as adapters, and their substrates and interacting partners are usually host proteins. However, some effectors can bind or modify host lipids; for instance: (i) SseJ has glycerophospholipid-cholesterol acyltransferase activity that is activated upon interaction with GTP-RhoA [13]. This leads to cholesterol esterification, which could alter the fluidity of the SCV membrane [14]. (ii) SteA specifically binds to phosphatidylinositol 4-phosphate [PI(4)P], and this binding is necessary for its localization at the SCV membrane and to *Salmonella*-induced tubules [15].

SrfJ is a T3SS2 effector the synthesis of which is positively regulated by SsrB [16], the main regulator of SPI2 [17], and negatively by IolR [18], the regulator of the *myo*-inositol utilization island [19]. This dual regulation is due to the existence of two distinct promoters that control the expression of the gene *srfJ* [20]. A proximal promoter, P*srfJ*, responds to intravacuolar signals and is positively regulated by SsrB and PhoP and negatively regulated by RcsB. A distal promoter, P*iolE*, responds to *myo*-inositol and is negatively regulated by IolR. The proximal promoter is active inside mammalian cells, while the distant promoter is expressed during *Salmonella*’s colonization of plants, which are alternative hosts where *myo*-inositol is a ubiquitous constituent [21]. Interestingly, inappropriate expression of *srfJ* leads to reduced proliferation within macrophages, and the lack of expression of *srfJ* decreases the activation of defense responses in tomato plants [20], suggesting that SrfJ could act as an avirulence protein [22] in this system. In addition, the virulence of a *S.* Typhimurium strain with a mutation in *srfJ* was mildly attenuated in mice [23].

The amino acid sequence of SrfJ is 30% identical to that of human glucosylceramidase on 447 residues and there are high structural similarities in the overall fold and the active-site environment of both proteins [24]. These similarities suggest that this effector may catalyze the hydrolysis of glucosylceramide into glucose and ceramide, a member of the family of sphingolipids. In addition to being structural elements of membranes, some members of this family, including ceramide, are secondary messengers that can affect important cellular processes, including proliferation, differentiation, cell death, oxidative stress, and inflammation [25].

Here, we explore the biochemical function of SrfJ and the consequences for mammalian cells of its expression and translocation. Our experiments suggest that SrfJ is a glucosylceramidase that significantly modifies the lipid profile of transfected epithelial cells and contributes to altering the expression of genes involved in processes related to immune response, cell communication, and cell death in infected macrophages.

## 2. Results

### 2.1. Glucosylceramidase Activity of SrfJ

**Figure 1:**
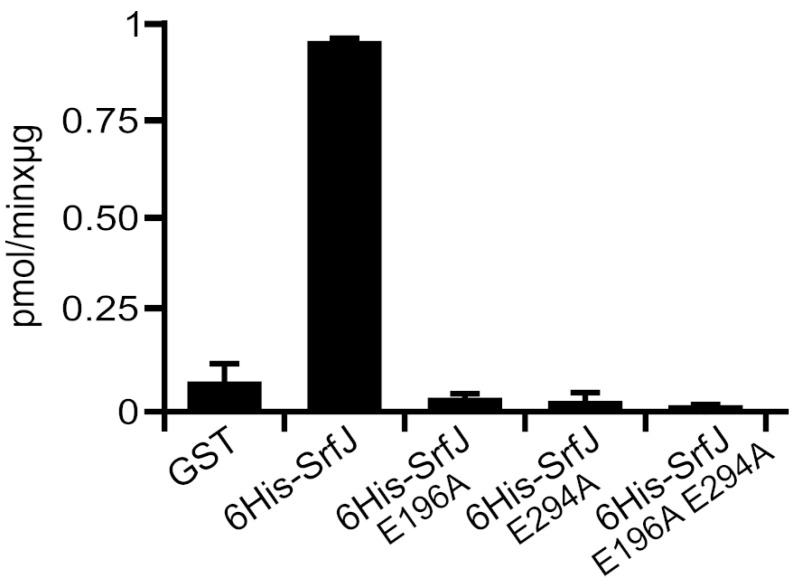
SrfJ is a glucosylceramidase. The activity of the indicated proteins was measured by their ability to hydrolyze 4-methylumbelliferyl-β-D-glucopyranoside. Specific activity was calculated as pmol of product produced per min and µg of protein using 4-methylumbelliferone as a calibration standard. Values are means + standard deviations. n = 3.

The similarity between SrfJ and human glucosylceramidase led us to explore the enzymatic activity of this *Salmonella* effector. For this purpose, the 4-methylumbelliferyl-β-D-glucopyranoside substrate was used. Hydrolysis of this substrate results in a product with emission at the blue region of the fluorescence spectrum. The purified 6His-SrfJ in this assay showed a glucosidase activity of 0.83 U/mg, significantly higher than the background hydrolysis obtained with the same amount of GST, used as a negative control (Figure 1). Similar results were obtained with an alternative substrate, resorufin β-D-glucopyranoside [26]. There are two glutamic acid residues in SrfJ, Glu196 and Glu294, which, according to the crystal structure of the protein [24], are located at positions equivalent to the catalytic residues Glu235 and Glu340 of human glucosylceramidase. To test the hypothesis that these residues are also relevant for the activity of SrfJ, three different mutants were obtained in the 6His fusion plasmid to generate the proteins 6His-SrfJE196A, 6His-SrfJE294A, and 6His-SrfJE196AE294A (Figure S1), in which one or both putative catalytic glutamic acid residues were changed into alanines. All these mutants completely lost activity since they produced the same level of fluorescence in the in vitro assay as the protein used as a negative control (Figure 1). These results, together with similarity data, suggest that SrfJ possesses glucosylceramidase activity and that Glu196 and Glu294 are essential for this function.

### 2.2. Effect of SrfJ on the Host Lipidome

**Figure 2:**
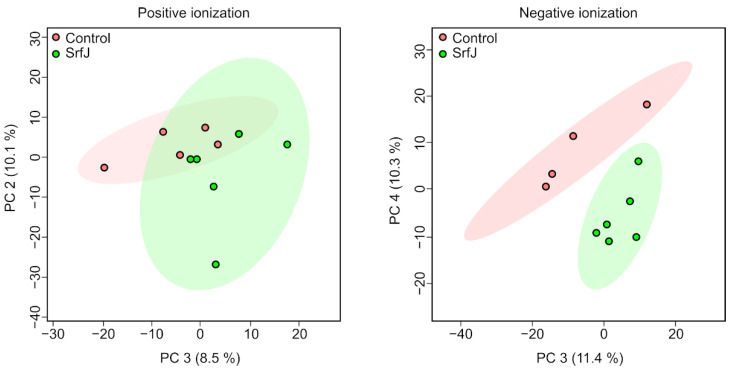
Alteration of the human cell lipidome induced by SrfJ. Principal component analysis score plot of lipids detected in positive ionization mode (**left** panel) and negative ionization mode (**right** panel) comparing HEK293T cells expressing SrfJ (green spots) with cells transfected with empty vector (red spots).

The in vitro activity shown for SrfJ in the previous section suggested that this *Salmonella* effector could have a role in the manipulation of lipids in the host cell. To test this hypothesis, we transfected human HEK293T cells either with a plasmid producing SrfJ or with the empty vector, and lipids were extracted and analyzed using high-throughput lipidome profiling mass spectrometry. This analysis detected 917 compounds in positive ionization mode and 933 in negative ionization mode. Multivariate statistics (principal component analysis) showed a clear alteration of the lipidome induced by the presence of SrfJ, with clear separation between control and SrfJ-expressing cells for lipids with negative ionization (Figure 2).

**Figure 3:**
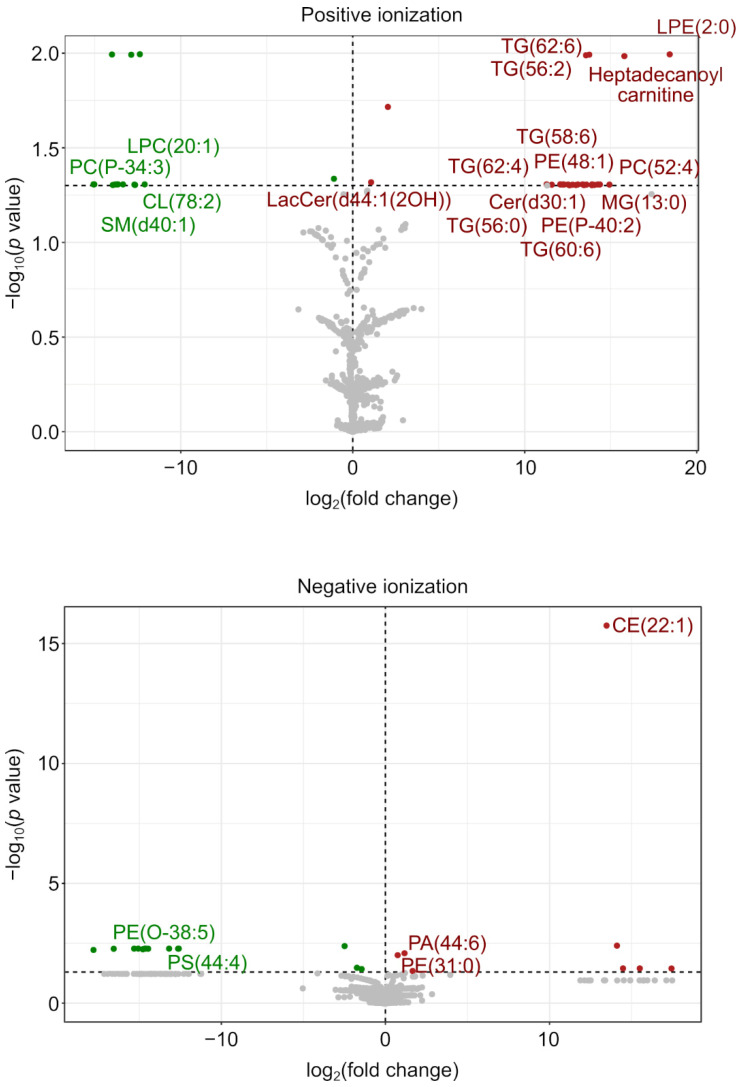
Effect of SrfJ on the lipidome of HEK293T cells. Volcano plots showing the log_2_ of the fold change in the abundance of lipid species detected in positive ionization mode (upper panel) or negative ionization mode (lower panel) in SrfJ-expressing vs. empty vector-transfected HEK293T cells (X-axis) plotted against the −log_10_ (*p* value) (Y-axis). Identified lipids are labeled as in Table 1. Red dots: lipids more abundant in the presence of SrfJ; green dots: lipids less abundant in the presence of SrfJ; grey dots: lipids not significantly differentially altered for *p* < 0.05.

Univariate statistics (*t*-test, *p* < 0.05) revealed 64 lipid species (42 in positive ionization mode and 22 in negative ionization mode) with significantly different levels between both conditions (Table S1). We were able to determine the identity of some of them based on exact mass, retention time, isotopic distribution, and MS/MS spectrum. This analysis identified seventeen lipids with higher levels and six lipids with lower levels in the presence of SrfJ (Table 1 and Figure 3).

**Figure 4:**
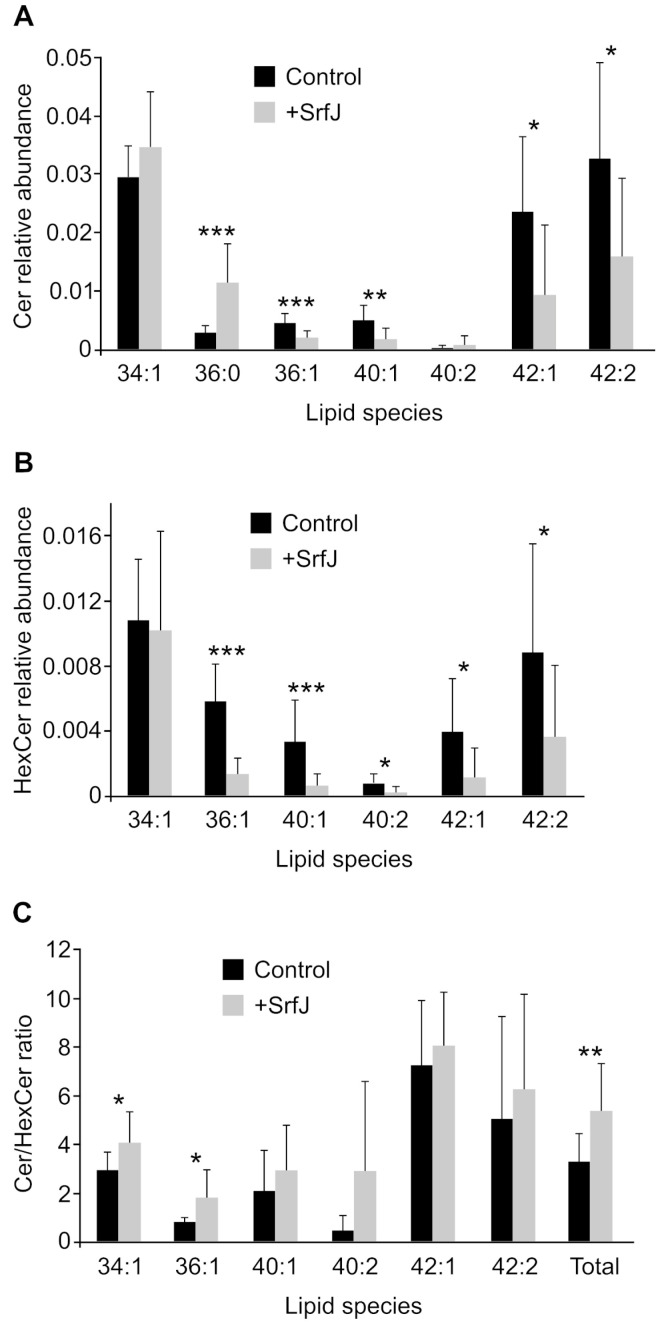
Effect of SrfJ expression on ceramide and hexosylceramide abundance and the ratios of ceramide/hexosylceramide in HEK293T cells. Relative abundance of ceramides (**A**) and hexosylceramides (**B**) in HEK293T cells transfected with an empty vector (Control) or with a plasmid expressing SrfJ (+SrfJ). (**C**) Ceramide/hexosylceramide ratios for 34:1, 36:1, 40:1, 40:2, 42:1, 42:2 species and for the addition of all these lipid species. Data are presented as mean values + standard deviations of ten replicates. Differences were considered significant if *p* < 0.05 (Student’s *t* test). * *p* < 0.05, ** *p* < 0.01, *** *p* < 0.001.

Interestingly, consistent with the glucosylceramidase activity of SrfJ, one of the lipids that was over-represented in the presence of SrfJ was a ceramide. Then, we decided to perform a targeted analysis of ceramides and hexosylceramides (which include glucosylceramides and galactosylceramides, that cannot be distinguished with these methods) comparing HEK293T cells transfected with pcDNA3 (empty vector) or pIZ1855 (pcDNA3-SrfJ-3xFLAG). This analysis revealed an impact of SrfJ on the levels of these sphingolipids (Figure 4A,B). Importantly, the Cer/HexCer ratio was increased in SrfJ-expressing cells, and this increase was statistically significant for the specific species 34:1 and 36:1, as well as for the total amount of these lipids (Figure 4C).

**Figure 5:**
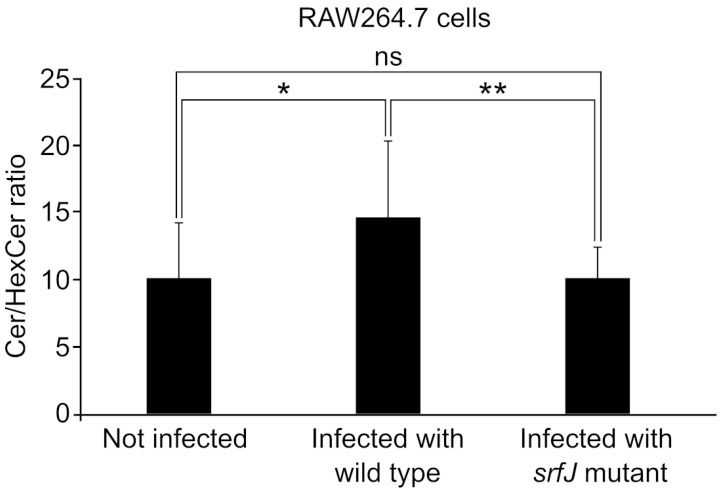
Effect of *Salmonella* infection on the ratios of ceramide/hexosylceramide in RAW264.7 macrophages. Total ceramide/hexosylceramide ratios in RAW264.7 macrophages not infected, infected with wild-type *S. enterica* serovar Typhimurium strain 14028, or infected with a Δ*srfJ* mutant. Data are presented as mean values + standard deviations of twelve biological replicates. Differences were considered significant if *p* < 0.05 (Student’s *t* test). * *p* < 0.05, ** *p* < 0.01, ns: not significant, for the indicated comparisons.

To obtain additional data on the effect of SrfJ on the host lipidome under physiological conditions, we infected RAW264.7 murine macrophages with either wild-type *Salmonella* or a Δ*srfJ* strain and analyzed the abundance of ceramides in these cells at 8 h post-infection and in uninfected cells. As seen in Figure 5, infection with *Salmonella* causes a decrease in glycosylated ceramides and an increase in the relative abundance of ceramides. Interestingly, the ratio Cer/HexCer is higher in wild-type infected cells than in cells infected with a *srfJ* mutant, suggesting that SrfJ plays a role in the generation of the altered patterns observed in host lipids.

### 2.3. Effect of SrfJ on the Host Transcriptome

**Figure 6:**
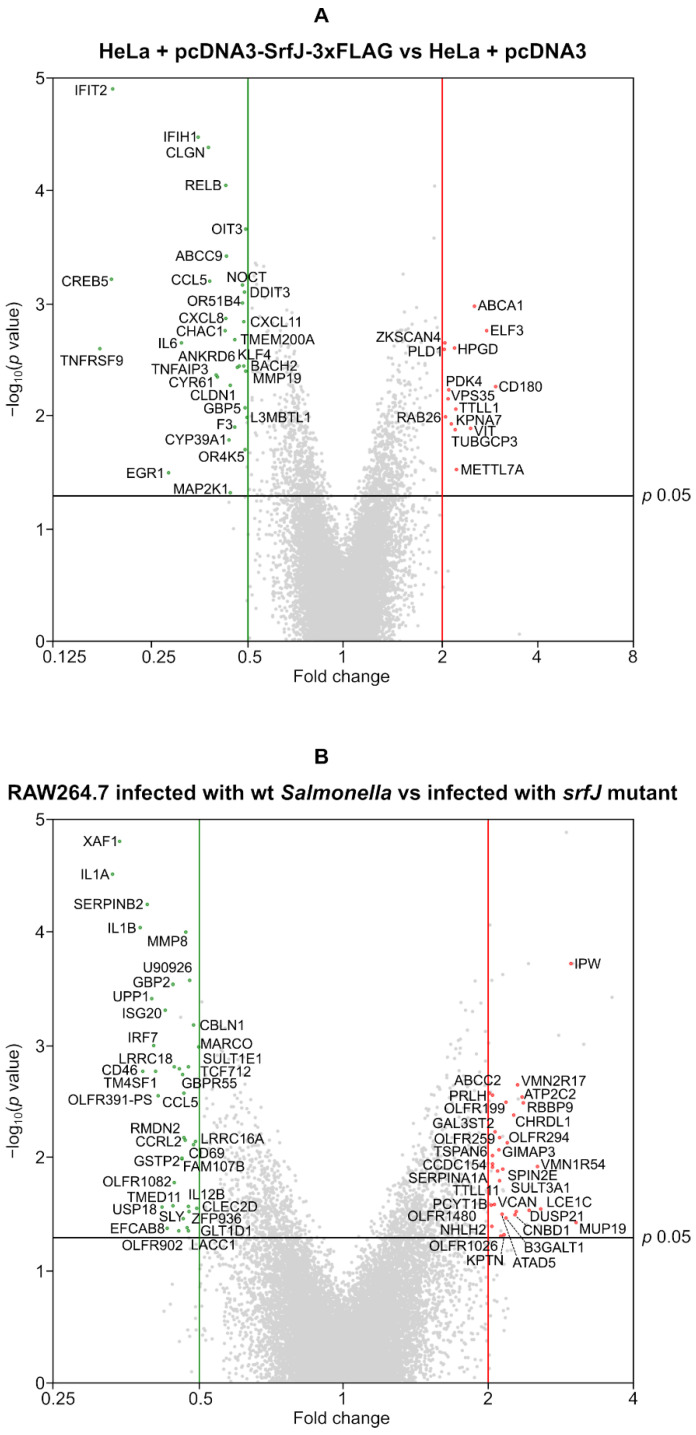
Effect of SrfJ on the transcriptome of host cells. (**A**) Volcano plots showing the fold change in the expression of genes in SrfJ-expressing vs. empty vector-transfected HeLa cells (X-axis) plotted against the −log_10_ (*p* value) (Y-axis). (**B**) Volcano plots showing the fold change in the expression of genes in RAW264.7 cells infected with wild-type *Salmonella* strain 14028 vs. RAW264.7 cells infected with a *srfJ* mutant (X-axis) plotted against the −log_10_ (*p* value) (Y-axis). Red dots: genes up-regulated in the presence of SrfJ; green dots: genes down-regulated in the presence of SrfJ; grey dots: genes not significantly differentially expressed for *p* < 0.05, that do not reach the fold-change threshold of 2 or 0.5, or that are predicted genes of unknown function.

Ceramides are involved in several important signal transduction pathways [27,28]. Therefore, we reasoned that alterations in the proportions of sphingolipids induced by SrfJ may also have an impact on the expression of host genes. To study this possibility, we performed a transcriptomic analysis using two different approaches. First, we analyzed the specific effect of the ectopic expression of SrfJ on epithelial human HeLa cells. RNA was obtained from HeLa cultures transiently transfected with either pcDNA3-SrfJ-3xFLAG or pcDNA3 (empty vector), and the analysis of their transcriptomes was performed using Clariom S Assay, HUMAN microarrays (Affymetrix, Santa Clara, CA, USA). Figure 6A represents the 1201 genes with statistically significant (*p* < 0.05) differential expression in SrfJ-transfected compared to HeLa cells transfected with the empty vector. The 45 differentially expressed genes with a linear fold change threshold of 2 or more for upregulated genes, 0.5 or less for downregulated genes, are described in Table S2. For a more physiological approach, we compared the transcriptomes of RAW264.7 murine macrophages infected for 8 h by either wild-type *Salmonella* or a *srfJ* mutant derivative, using Clariom S Assay, MOUSE microarrays (Affymetrix, Santa Clara, CA, USA) to compare transcriptomes. Genes with statistically significant (*p* < 0.05) differential expression in this comparison are represented in Figure 6B. Among 1408 differentially expressed genes, 68 overcame a linear fold change threshold of 2 for upregulated genes, 0.5 for downregulated genes (excluding predicted genes of unknown function). These genes are described in Table S3.

### 2.4. Functions of Genes Differentially Expressed in the Presence of SrfJ

**Figure 7:**
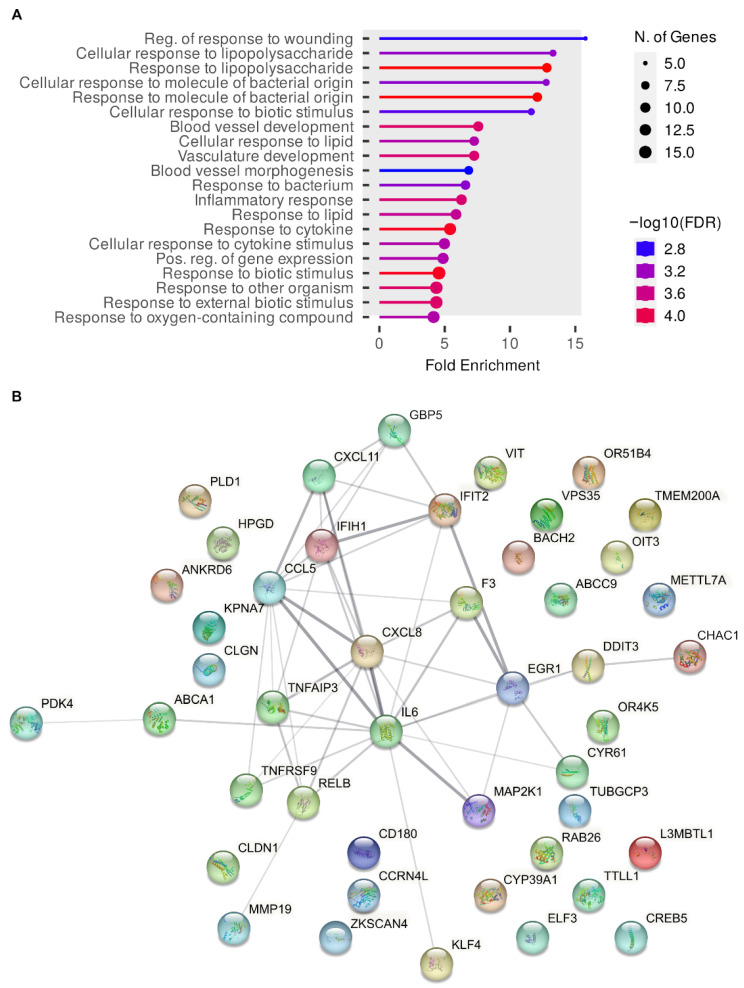
Gene ontology and network analyses of differentially expressed genes in HeLa cells transfected with a plasmid expressing SrfJ. (**A**) Enrichment analysis was carried out using the web application ShinyGO. The top 20 significantly enriched terms sorted by fold enrichment are shown. Colors indicate the levels of significance, and circle sizes are proportional to the number of differentially expressed genes (N. of genes) belonging to a pathway, as indicated in the legends. (**B**) Network of protein-protein interactions detected using the STRING database. The thickness of the line indicates the strength of the data support for each interaction.

To gain insight into cell processes affected by SrfJ, we explored the sets of differentially expressed genes using ShinyGO version 0.77 [29]. This analysis detected a significant enrichment of gene ontology (GO) terms in the database of biological processes for the set of genes that were differentially expressed in HeLa cells producing SrfJ (Figure 7A). Among the processes involved are the inflammatory response and the response to different stimuli, including molecules of bacterial origin. The network of interactions between proteins encoded by these genes was obtained using STRING [30] and is depicted in Figure 7B.

**Figure 8:**
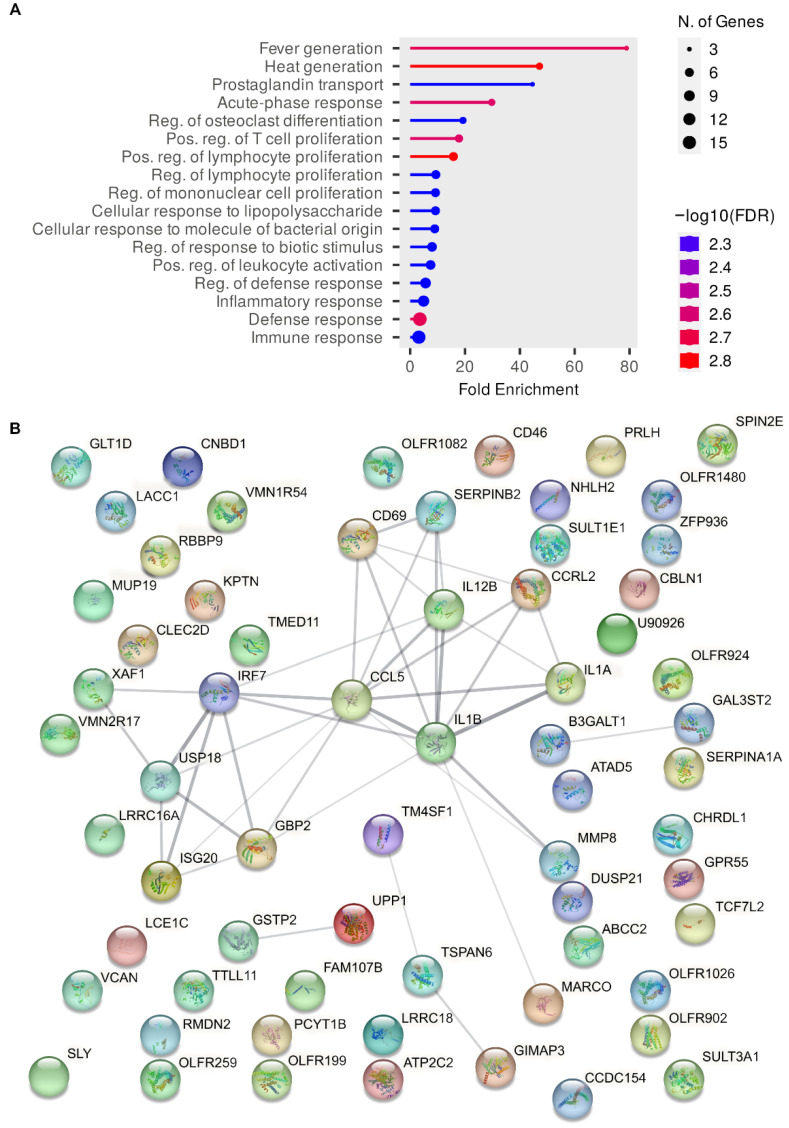
Gene ontology and network analyses of differentially expressed genes in RAW264.7 cells infected with wild-type versus *srfJ* mutant *Salmonella*. (**A**) Enrichment analysis was carried out using the web application ShinyGO. Significantly enriched terms are shown sorted by fold enrichment. Colors indicate the levels of significance, and circle sizes are proportional to the number of differentially expressed genes (N. of genes) belonging to a pathway, as indicated in the legends. (**B**) Network of protein-protein interactions detected using the STRING database. The thickness of the line indicates the strength of the data support for each interaction.

A similar analysis showed that some biological processes were overrepresented in the set of differentially expressed genes in the comparison between RAW264.7 cells infected with wild-type *Salmonella* and RAW264.7 cells infected with the *srfJ* mutant (Figure 8).

Interestingly, some common processes were detected to be enriched in both sets of differentially expressed genes, including the inflammatory response and the response to different stimuli.

### 2.5. Secretion of CCL5 by Host Cells Is Modulated by SrfJ

**Figure 9:**
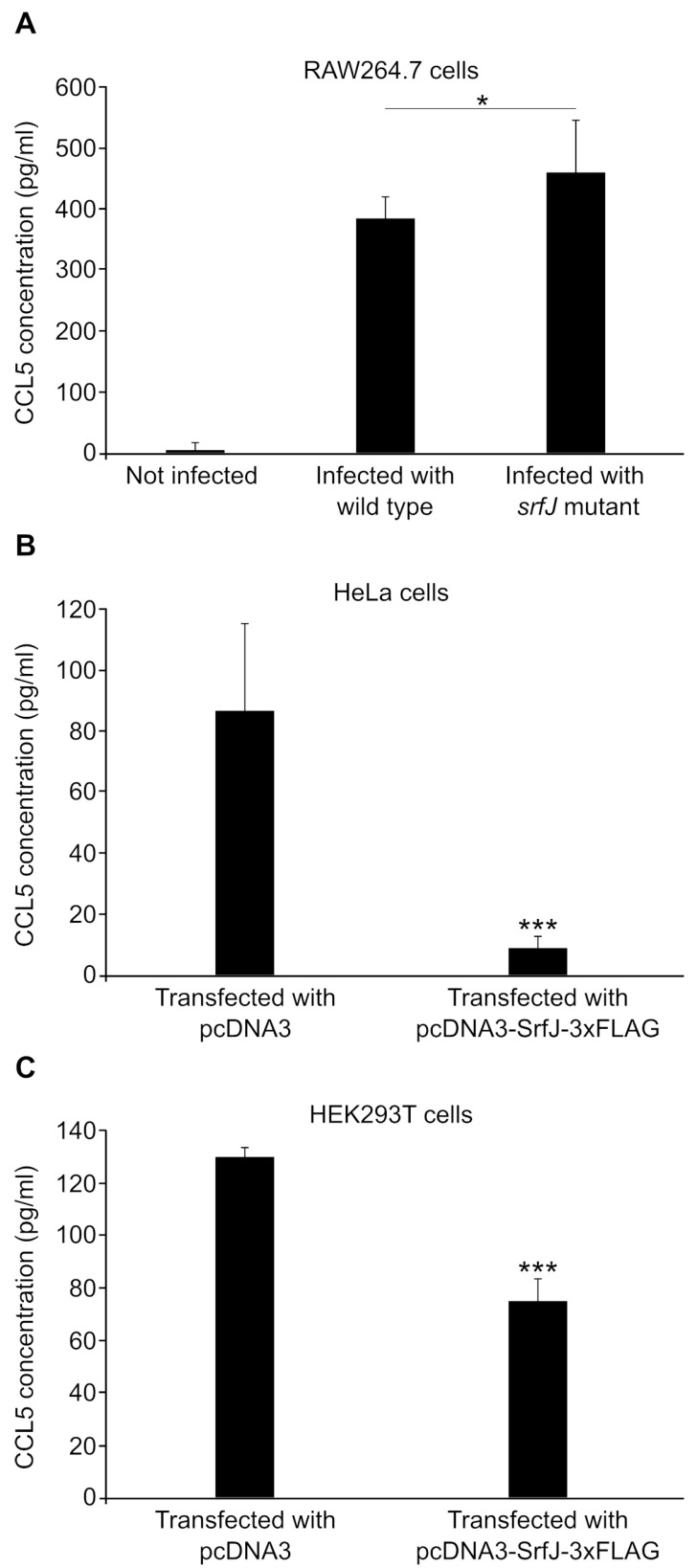
Effect of SrfJ on the secretion of CCL5 by host cells. (**A**) Murine RAW264.7 macrophages were infected with wild-type *Salmonella* strain 14028 or a Δ*srfJ* mutant. The concentration of CCL5 was measured in the supernatants of non-infected cells and cells infected for 8 h. Human HeLa cells (**B**) or HEK293T cells (**C**) were transfected with a plasmid expressing SrfJ (pcDNA3-SrfJ-3xFLAG) or with the empty vector (pcDNA3) and the concentration of CCL5 was measured in the supernatants of cell cultures 24 h after transfection. Data are presented as mean values + standard deviations of six biological replicates. * *p* < 0.05, *** *p* < 0.001, for a one-tailed Student’s *t*-test.

The only gene the expression of which changed more than two-fold in the presence of SrfJ both in transfected HeLa cells and in infected RAW264.7 cells was *CCL5*. The product of this gene, CCL5, also known as RANTES, is a chemokine that induces leukocyte migration and can mediate inflammatory responses [31]. To explore the possibility that SrfJ may modulate the secretion of CCL5 in host cells, we infected RAW264.7 macrophages with wild-type *S. enterica* serovar Typhimurium or with a Δ*srfJ* mutant and measured the concentration of the chemokine in the supernatants of cell cultures 8 h post-infection. As seen in Figure 9A, *Salmonella* infection triggers the secretion of CCL5 by macrophages as expected, but the level of secretion increases when infecting with an Δ*srfJ* mutant, suggesting that SrfJ contributes to the modulate secretion of this chemokine. To confirm the effect of SrfJ on CCL5 secretion, human HeLa cells and HEK293T cells were transfected with a plasmid expressing SrfJ or with the empty vector as a control. The results shown in Figure 9B,C indicate that SrfJ very significantly decreases the secretion of CCL5.

## 3. Discussion

Most T3SS effectors target host proteins and there have been many studies describing the roles of these interactions in *Salmonella* virulence. On the contrary, studies relating effectors to host lipids are scarce. However, the subversion of host lipid signaling is essential for successful infection [32]. Phosphoinositides are particularly important during phagosome maturation, and *Salmonella* manipulates their metabolism through the T3SS effector SopB. This effector functions as a phosphoinositide phosphatase and phosphotransferase [33]. Indeed, there have been reports showing that *S. enterica* serovar Typhimurium infection results in an increase in intracellular cholesterol content in a T3SS2-dependent manner. For instance, the effectors SseJ and SseL have roles in cholesterol metabolism and transport. SseJ is a RhoA-dependent cholesterol acyltransferase, the activity of which is necessary to suppress the expression of the gene encoding the ABCA1 transporter through a signaling cascade that involves FAK and Akt kinases [34]. Both SseJ and SseL interact with the host cholesterol transport protein OSBP1 [35,36].

SrfJ shares 30% amino acid sequence identity with human glucosylceramidase on its 447 residues, and their structures are highly similar [24]. These similarities suggested that SrfJ could also be a glucosylceramidase. Here, using an in vitro assay, we have shown that SrfJ indeed possesses this activity, also showing the essential role of the predicted catalytic residues Glu196 and Glu294 (Figure 1). To the best of our knowledge, this is the first T3SS effector shown to have glucosylceramidase activity. The expression of *srfJ* causes significant changes in the lipidome of human cells, which are compatible with the activity observed in vitro, since the ratios HexCer/Cer are increased (Figure 4), suggesting that SrfJ is also active as a glucosylceramidase in vivo. Importantly, these results are confirmed in the context of host cell infections. *Salmonella* infection increases the HexCer/Cer ratio of murine macrophages in a SrfJ-dependent manner (Figure 5). The effect of SrfJ in this context is not related to differential intracellular survival, since our previous results showed that the *srfJ* mutant has no significant defect in intracellular proliferation compared to the wild-type strain [20]. In addition to Cer and HexCer, the abundance of many other lipids is altered by the presence of SrfJ, which could be due either to indirect effects or to other unknown activities of this effector.

Ceramide is the precursor of all complex sphingolipids. These are important signaling molecules that regulate numerous physiological and pathological processes, including signal transduction, the regulation of cell growth and death, adhesion, migration, immunity, and inflammation [37,38]. Bioactive sphingolipids are involved in the regulation of the internalization of bacteria in the host cell and the induction of an immunological response. However, some bacterial pathogens are capable of interfering with sphingolipid signaling by hijacking specific host cell factors or producing enzymes that are involved in sphingolipid metabolism [39]. Examples of the latter are sphingomyelinases of *Bacillus cereus, Staphylococcus aureus, Listeria ivanovii, Mycobacterium tuberculosis* and *Legionella pneumophila*, ceramidase and sphingomyelin synthase of *Pseudomonas aeruginosa*, sphingosine phosphate lyase of *Burkholderia pseudomallei, Burkholderia thailandensis* and *L. pneumophila*, and sphingosine kinase from *L. pneumophila*.

Since ceramide and other derived sphingolipids are involved in the control of several signaling pathways that may ultimately affect the transcription of host genes, we decided to explore the effects of SrfJ on the host transcriptome. This analysis revealed that the levels of transcriptional products of many genes are significantly altered by SrfJ both when expressed alone in epithelial cells and in the context of macrophage infections (Figure 6, Tables S2 and S3). Interestingly, the enrichment analysis points to genes involved in the innate immune response and inflammation among those modulated by SrfJ (Figure 7A and Figure 8A). Importantly, this analysis was validated by our experiments, showing a small but significant increase in the secretion of the chemokine CCL5 from macrophages that were infected with a *srfJ* mutant strain of *Salmonella* compared to those that were infected with the wild-type strain (Figure 9A). These results were confirmed by transfection experiments in which the presence of SrfJ very significantly reduced the secretion of CCL5 in human cells (Figure 9B,C). This cytokine functions as a chemoattractant for blood monocytes, memory T helper cells, and eosinophils, causing the release of histamine from basophils, and activating eosinophils [40]. Our results suggest that SrfJ may contribute to a moderation of the level of host cytokine secretion induced by *Salmonella* infection, and thus protect bacteria from host immune defenses.

In summary, this work reveals for the first time the existence of a T3SS effector with glucosylceramidase activity, the presence of which in host cells results in significant alterations of lipid patterns and gene expression that may have immunomodulatory consequences in the host.

## 4. Materials and Methods

### 4.1. Bacterial Strains and Plasmids

The microbial strains and plasmids used in this study are described in Table 2. *S.* Typhimurium strains derived from the wild-type strain ATCC 14028. Transductional crosses using the phage P22 HT 105/1 int201 [41] were used for the construction of *Salmonella* strains [42].

### 4.2. DNA Amplification with Polymerase Chain Reaction and Sequencing

Amplification reactions were carried out on a T100 Thermal Cycler (Bio-Rad, Hercules, CA, USA) using Q5 High-Fidelity DNA polymerase (New England Biolabs, Ipswich, MA, USA) or MyTaq Red DNA polymerase (Bioline Memphis, TN, USA) according to the supplier’s instructions. Oligonucleotides are described in Table 3. The constructs were sequenced with an automated DNA sequencer (Stab Vida, Oeiras, Portugal).

### 4.3. Bacterial Culture

The standard culture medium for *S. enterica* and *Escherichia coli* was LB broth. Solid LB contained agar 1.5% final concentration. Antibiotics were used at the following concentrations: kanamycin (Km), 50 μg/mL; ampicillin (Ap), 100 μg/mL.

### 4.4. Mutagenesis

The disruption of *srfJ* and the replacement with a gene conferring Km resistance was carried out as previously described [45] using primers srfJP1 and srfJP2 and the plasmid pKD13. The antibiotic resistance cassette introduced by the gene targeting procedure was eliminated by recombination using the FLP helper plasmid pCP20.

To generate a point mutation that affects the putative catalytic residue Glu196 of SrfJ, the plasmid pIZ2046 (pQE30-SrfJ) was used as a template for PCR amplification using the primer pair srfJE196Afw/srfJE196Arv. The products were digested with 1 μL of DpnI (10 U/μL) for 1 h at 37 °C and used to transform *E. coli* XL1-Blue. Overlap extension PCR was used to obtain a similar mutation that affects residue Glu294 using primer pairs srfJBamfw/srfJE294Arv, srfJE294Afw/srfJSalrv, and srfJBamfw/srfJSalrv. The final product was digested with BamHI and SalI and ligated to pQE30 digested with the same endonucleases. Mutations were verified by Sanger sequencing.

### 4.5. GST and 6His Fusion Proteins

GST expression was induced by adding 1 mM isopropyl-β-D-thiogalactoside to bacteria containing pGEX-4T-1, and the protein was isolated from bacterial lysates by affinity chromatography with glutathione-agarose beads (Sigma-Aldrich, San Luis, MO, USA). For lysis, the bacteria were sonicated in NP40 buffer. Then, 6His fusion proteins were produced after the addition of 1 mM isopropyl-β-D-thiogalactoside to *E. coli* XL1-Blue containing derivatives of pQE30, purified in Ni-NTA agarose beads (Sigma-Aldrich), and eluted with 300 mM imidazole in binding buffer (50 mM NaH_2_PO_4_, 300 mM NaCl).

### 4.6. Glucosylceramidase Assay

The activity of the recombinant proteins was measured by their ability to hydrolyze 4-methylumbelliferyl-β-D-glucopyranoside [46,47]. The SrfJ proteins (wild-type and mutants) were purified as 6His fusion proteins and diluted in assay buffer (50 mM sodium citrate, 25 mM sodium cholate, 5 mM DTT, pH 6.0). The reaction was started by mixing 25 μL of the diluted proteins with 25 μL of 6 mM substrate (4-methylumbelliferyl-β-D-glucopyranoside) prepared in assay buffer. After 20 min of incubation at 37 °C, the reaction was stopped by adding 50 μL of stop solution (0.5 M glycine, 0.3 M NaOH, pH 10.0). Fluorescence was measured at excitation and emission wavelengths of 365 nm and 445 nm (top read), respectively, in endpoint mode. Specific activity was calculated as pmol of product produced per min and μg of protein using 4-methylumbelliferone as a calibration standard.

### 4.7. Cell Culture, Lysis, and Transfection

HeLa (human epithelial; ECACC no. 93021013), HEK293T (human embryonic kidney SV40 transformed; ECACC no. 12022001), and RAW264.7 (murine macrophages; ECACC no. 91062702) cells were cultured in DMEM supplemented with 10% fetal calf serum. Then, 2 mM L-glutamine, 100 U/mL penicillin, and 100 μg/mL streptomycin were included in the culture medium. All cells were kept in a humidified atmosphere with 5% CO_2_ at 37 °C. For cell lysis, 2 × 10^7^ to 10^8^ cells per ml were incubated at 4 °C in NP40 buffer (10 mM Tris-HCl pH 7.4, 150 mM NaCl, 10% glycerol, 1% NP40, 1% aprotinin, 1 mM PMSF, 1 μg/mL pepstatin, and 1 μg/mL leupeptin) for 20 min. The extract was centrifuged at 20,000× *g* for 20 min and the supernatant was stored at −80 °C. For transient transfection assays, 2–5 × 10^6^ HeLa cells/assay were resuspended in 200 μL of 15 mM Hepes-buffered serum-containing medium, mixed with 50 μL of 210 mM NaCl containing 5–10 μg plasmid DNA and electroporated using a BTX Electrocell Manipulator 600 set at 240 V, 720 Ω, 950 μF. Cells were processed 24 h after electroporation. This protocol yielded an efficiency of about 30–40% of transfected cells. HEK293T cells were transfected using Xfect reagent (Takara) according to the manufacturer’s instructions to obtain a percentage of transfection of around 80%. No significant differences in viability were observed between cells transfected with pcDNA3 or pcDNA3-SrfJ-3xFLAG.

### 4.8. Infections of RAW264.7 Cells with Salmonella

RAW264.7 cells were seeded in 24-well plates at 1.5 × 10^5^ cells per well and incubated for 24 h at 37 °C with 5% CO_2_. The bacteria were grown in LB medium for 24 h at 37 °C with shaking and added at a multiplicity of infection of 250. Cell culture was washed twice with phosphate buffered saline (PBS) 1 h after infection, overlaid with DMEM containing 100 μg/mL gentamicin, and incubated for another hour. The culture was then washed twice with PBS, covered with DMEM with gentamicin (16 μg/mL), and incubated for 6 h. Infected RAW264.7 cells showed a 65% viability compared to non-infected cells. This viability was similar using the *srfJ* strain and wild-type *Salmonella*.

### 4.9. Lipidomic Analysis

Untargeted lipidomic analysis was carried out at the Lipidomics Platform of the Biomedical Research Institute of Lleida (Lleida, Spain). Lipids were extracted from transfected HEK293T cells (one million cells per sample, six samples per condition) using an MTBE-based method, as previously described [48]. Lipid extracts were subjected to liquid chromatography coupled to mass spectrometry (LC-MS) using an Agilent UPLC 1290 coupled to the Q-TOF MS/MS 6520 (Agilent Technologies, Santa Clara, CA, USA) based on previously published methods [49,50]. Duplicate runs of each sample were performed to collect positive and negative electrospray ionized lipid species. The identity of relevant lipid species was confirmed by exact mass, retention time, and/or MS/MS spectrum, as previously described [51].

The targeted lipidomic analysis was performed by the Biomolecular Mass Spectrometry (BIO-MS) service of the University Pablo de Olavide (Seville, Spain). For this analysis, each cell pellet was resuspended in 1 mL of MeOH/MTBE/CHCl_3_ (1.33:1:1, *v*/*v*/*v*, MTBE stands for methyl tert-butyl ether) with added internal standards (Cer(35:1), HexCer(35:1), Hex2Cer(35:1)), and vortexed for 20 s. Subsequently, the mixture was incubated in a shaker at 900 rpm for 1 h at room temperature (RT). The sample was then sonicated for 30 min and incubated for 20 min at RT. Particulate matter was pelleted by centrifugation at 16,000× *g* for 10 min at 20 °C. The supernatant was collected and dried in a vacuum centrifuge for 120 min at 30°C. The extracted lipids were reconstituted in 60 µL butanol:water (1:1, *v*/*v*) and sonicated for 10 min. Then, 60 µL of methanol with 10 mM ammonium formate was added and the samples were centrifuged (1711 g, 6 min, 20 °C). The supernatants were then transferred to a 0.2 mL glass vial for UHPLC-MS/MS analysis. Lipidomic analysis was performed by liquid chromatography-electrospray ionization-tandem mass spectrometry (LC-ESI-MS/MS) using a Thermo Scientific-Dionex Ultimate 3000 UHPLC coupled to a Q Exactive™ Hybrid Quadrupole-Orbitrap High Resolution Mass Spectrometer. The lipids were separated by reversed-phase chromatography using a Zorbax Eclipse Plus C18 column (2.1 × 50 mm, 1.8 µm, Agilent Technologies, Santa Clara, CA USA). Solvents A and B consisted of tetrahydrofuran:methanol:water in ratios 20:20:60 and 75:20:5, respectively, both containing 10 mM ammonium formate. The column and autosampler temperatures were regulated at 50 and 25 °C, respectively. The following gradient conditions were used: (flow rate 400 μL/min) 0% solvent B to 40% solvent B for 2 min, 40% solvent B to 100% solvent B for 6.5 min, 0.5 min at 100% solvent B, and a return to 0% solvent B for 0.5 min, and then 0.5 min at 0% solvent B prior to the next injection. The liquid chromatography eluent was then analyzed by ESI-MS/MS using full MS/ddMs^2^ scan mode. Mass spectrometry detection was performed using a Q Exactive™. Lipids were detected by electrospray ionization in positive mode with a mass resolution of 70,000. Nitrogen was used as a desolvation gas. Data were acquired in the m/z range from 150 to 1500. Electrospray ionization parameters were as follows: sheat gas flow 45 arb (arbitrary units), auxiliary gas flow 8 arb, auxiliary gas heater temperature 350 °C, spray voltage 3.5 kV, capillary temperature 320 °C.

For statistical analyses of lipidomic analyses, Mass-Hunter Mass Profiler Professional software (Agilent Technologies, Santa Clara, CA, USA) and Metaboanalyst software version 5.0 [52] were used. Other statistical calculations were performed using SPSS software version 26 (Chicago, IL, USA). Differences in the number of molecules between conditions were analyzed using a *t*-test. A level of *p* < 0.05 was considered statistically significant.

### 4.10. RNA Preparation, Gene Array Processing, and Statistics

Total RNA from HeLa or RAW264.7 cells was isolated using 1 mL of TRIzol reagent (Invitrogen) according to the manufacturer’s instructions. An additional purification step was performed with the RNeasy Min Elute Cleanup Kit (Qiagen, Hilden, Germany). Biotinylated single-stranded cDNA was prepared from 100 ng per sample of total intact RNA (3 samples per condition). Labeled cDNA was hybridized with the Clariom S Assay, HUMAN arrays (for HeLa RNA) or Clariom S Assay, MOUSE arrays (for RAW264.7 RNA) (Affymetrix) following the manufacturer’s instructions. The arrays were scanned on a 3000 7G scanner (Affymetrix). Image analysis, fluorescent data quantification, and quality control were performed with Affymetrix software, the GeneChip Command Console version 2.0, and the Transcriptome Analysis Console version 4.0.2. All procedures and preliminary data analysis were performed in the Genomics Unit of the Andalusian Center for Molecular Biology and Regenerative Medicine (CABIMER, Seville, Spain). Statistical significance (*p*-value) was calculated using the empirical Bayes moderated t test based on the results of three arrays per condition. The microarray data used in this analysis are available from the NCBI Gene Expression Omnibus at http://www.ncbi.nlm.nih.gov/geo/ (accessed on 7 October 2022) under accession numbers GSE215051 and GSE215053.

### 4.11. Detection of CCL5 Secretion by ELISA

Supernatants of murine RAW264.7 cells either non-infected or infected with wild-type or a *srfJ* mutant *Salmonella*, as well as supernatants of transfected human HeLa or HEK293T cells, were assayed for CCL5 secretion using the mouse or the human CCL5/RANTES DuoSet ELISA kits (R&D SYSTEMS) following manufacturer’s instructions. Briefly, 96-well polysorbent plates previously incubated overnight at room temperature with mouse CCL5 capture antibody were washed, and then blocked with Reagent Diluent for 2 h at room temperature and washed again. Then, 100 µL of appropriately diluted samples of supernatant (and serial dilutions of an appropriate mouse CCL5 standard, for CCL5 quantification) were transferred to the plates and incubated for 2 h at room temperature. Then, plates were washed again, followed by 2 h of incubation at room temperature with CCL5 detection antibody, a new wash, and 20 min of incubation, at room temperature and in the dark, with streptavidin-HRP B. Plates were then washed one final time, and incubated for 20 min at room temperature in the dark with substrate solution (TMB ELISA Substrate, R&D), upon which STOP solution was added, and signal development and detection were carried out using a Synergy HT plate reader (Biotek, Winooski, VT, USA) at a wavelength of 450 nm, with a subtraction wavelength of 570 nm. Serially diluted CCL5 standards were used for the extrapolation of protein concentration from absorbance data.

## Figures and Tables

**Table 1:** Statistically significant differences in lipid species induced by SrfJ in HEK293T cells.

Lipid Family (LIPID MAPS Classification)	Compound	Fold Change	*p* Value	Regulation in the Presence of SrfJ
Fatty acyls	Heptadecanoyl carnitine	5.67 × 10^4^	1.04 × 10^−2^	Up
Glycerolipids	MG (13:0)	1.54 × 10^4^	4.93 × 10^−2^	Up
	TG (56:0)	4.33 × 10³	4.94 × 10^−2^	Up
	TG (56:2)	1.21 × 10^4^	1.03 × 10^−2^	Up
	TG (58:6)	1.11 × 10^4^	4.98 × 10^−2^	Up
	TG (60:6)	7.03 × 10³	4.95 × 10^−2^	Up
	TG (62:4)	5.87 × 10³	4.95 × 10^−2^	Up
	TG (62:6)	1.38 × 10^4^	1.02 × 10^−2^	Up
Glycerophospholipids	LPE (2:0)	3.52 × 10^5^	1.01 × 10^−2^	Up
	PA (44:6)	2.24	8.25 × 10^−3^	Up
	PC (52:4)	1.71 × 10^4^	4.96 × 10^−2^	Up
	PE (31:0)	1.68	9.91 × 10^−3^	Up
	PE (48:1)	1.24 × 10^4^	4.95 × 10^−2^	Up
	PE (P-40:2)	1.14 × 10^4^	4.96 × 10^−2^	Up
Sphingolipids	Cer (d30:1)	6.22 × 10³	4.99 × 10^−2^	Up
	LacCer (d44:1(2OH))	2.09	4.81 × 10^−2^	Up
Sterol lipids	CE (22:1)	1.14 × 10^4^	1.78 × 10^−16^	Up
Glycerophospholipids	CL (78:2)	1.55 × 10^−4^	4.97 × 10^−2^	Down
	LPC (20:1)	6.89 × 10^−5^	4.94 × 10^−2^	Down
	PC (P-34:3)	2.89 × 10^−5^	4.93 × 10^−2^	Down
	PE (O-38:5)	2.45 × 10^−5^	5.30 × 10^−3^	Down
	PS (44:4)	1.62 × 10^−4^	5.29 × 10^−3^	Down
Sphingolipids	SM (d40:1)	6.29 × 10^−5^	4.97 × 10^−2^	Down

CE: cholesterol ester; Cer: ceramide; CL: cardiolipin; LacCer: lactosylceramide; LPC: lysophosphatidylcoline; LPE: lysophosphatidyletanolamine; MG: monoacylglycerol; PA: phosphatidic acid; PC: phosphatidylcholine; PE: phosphatidyletanolamine; PS: phosphatidylserine; SM: sphingomyelin; TG: triacylglycerol.

**Table 2:** Bacterial strains and plasmids used in this study.

Strain/Plasmid	Relevant Characteristics	Source/Reference
*Escherichia coli*		
BL21(DE3)	F^-^ *ompT gal dcm lon hsdS_B_* (r^−^ m^−^; *E. coli* B strain), with DE3, a λ prophage carrying the T7 RNA *pol* gene	Stratagene
DH5α	*supE44* ∆*lacU*169 (Ø80 *lacZ*∆M15) *hsdR17 recA1 endA1 gyrA96 thi-1 relA1*	[43]
XL1-Blue	*recA1 endA1 gyrA96 thi-1 hsdR17 supE44 relA1 Dlac-pro/*F’ *proAB lacI^q^ lacZ*DM15 Tn*10* (Tet^r^)	[44]
*Salmonella enterica* serovar Typhimurium		
14028	Wild type	ATCC
SV5599	*srfJ*::3XFLAG Km^r^	[18]
SV9689	Δ*srfJ*	This work
*Plasmids*		
pGEX-4T-1	GST fusion vector, Ap^r^	GE Healthcare
pIZ1855	pcDNA3-SrfJ-3xFLAG	This work
pIZ2046	pQE30-SrfJ	This work
pIZ3397	pQE30-SrfJ(E196A)	This work
pIZ3411	pQE30-SrfJ(E294A)	This work
pIZ3412	pQE30-SrfJ(E196A/E294A)	This work
pIZ3633	pcDNA3-SrfJ(E294A)-3xFLAG	This work

Tet^r^: tetracycline resistance, Km^r^: kanamycine resitance, Ap^r^: ampicillin resistance

**Table 3:** Oligonucleotides used in this study.

Oligonucleotide/ Use	Sequence 5’–3’
Deletion of *srfJ*	
srfJP1	cagatcgactcctgccgccatagcaacgtactggcgcctgGTGTAGGCTGGAGCTGCTTC
srfJP4	accgcgccacgcgttacagcagattgacggattcggcggcATTCCGGGGATCCGTCGACC
Construction of pIZ1855	
srfJpcdnadir	gtcaGGATCCgccaccATGAAAGGCAGACTCATCTC
3×FlagEcorev	ctgagaattcTTACTATTTATCGTCGTCATC
Construction of pIZ2046	
srfJBamfw	CTAGGGATCCAAAGGCAGACTCATCTCTTC
srfJSalrv	ATCTGTCGACAATCCCAGCTTCATCATTCAG
Construction of pIZ2397	
PsrfJKpnIfw	ATGCGGTACCTCACTGCGATGTTACCGGCG
srfJsalIrev	GTACGTCGACGATCGACTCCTGCCGCCATAG
Construction of pIZ3397	
srfJE196Afw	GCGCTCTCCGTGCAGAATGCGCCGGTGGCGGTAAAAACC
srfJE196Arv	GGTTTTTACCGCCACCGGCGCATTCTGCACGGAGAGCGC
Construction of pIZ3411 and pIZ3412	
srfJE294Afw	GATAAAAAACTCCTGTTTTCCGCGGGCTGTGTGCCAATGGAGAGC
srfJE294Arv	GCTCTCCATTGGCACACAGCCCGCGGAAAACAGGAGTTTTTTATC

## Data Availability

The data presented in this study are openly available from NCBI Gene Expression Omnibus at http://www.ncbi.nlm.nih.gov/geo/ (accessed on 7 October 2022) under accession numbers GSE215051 and GSE215053.

## Supplementary Materials

The following supporting information can be downloaded at: https://www.mdpi.com/article/10.3390/ijms24098403/s1.
