# Supplementary material for: Salmonella Type III Secretion Effector SrfJ: A Glucosylceramidase Affecting the Lipidome and the Transcriptome of Mammalian Host Cells
Source: Int J Mol Sci. 2023 May 7;24(9):8403. doi: 10.3390/ijms24098403 (PMC10179164; doi:10.3390/ijms24098403)
Supplement: Supplementary file 1 [file ijms-24-08403-s001.zip › ijms-2353762-supplementary.pdf]

Salmonella type III secretion effector SrfJ: a glucosylceramidase affecting the lipidome and the transcriptome of mammalian host cells

Julia Aguilera-Herce, Concepción Panadero-Medianero, María Antonia Sánchez-Romero, Roberto Balbontín, Joaquín Bernal-Bayard, Francisco Ramos-Morales

Supplementary files

Table S1. Lipid species with significantly different levels in the presence of SrfJ

p value	Fold change	Mean (SD) Control	Mean (SD) SrfJ	Mass	Retention time	ID
1.78E-16	11400	1.00 (0.00)	11400 (926)	688.6525	9.33	CE(22:1)
0.0499	6219.7	1.00 (0.00)	6220 (8242)	1025.8927	10.07	Cer(d30:1)
0.0497	0.00015461	6468 (7852)	1.00 (0.00)	1567.1508	7.25	CL(78:2)
0.0104	56672	1.00 (0.00)	56672 (69902)	435.3346	4.97	Heptadecanoyl carnitine
0.0481	2.0913	57908 (64327)	121103 (18838)	1025.2775	8.27	LacCer(d44:1(2OH))
0.0494	0.0000689	14510 (16613)	1.00 (0.00)	531.3772	0.9	LPC(20:1)
0.0101	352400	1.00 (0.00)	352403 (293808)	331.3229	2.36	LPE(2:0)
0.0493	15420	1.00 (0.00)	15420 (17045)	288.3026	0.93	MG(13:0)
0.00825	2.2399	11891 (23780)	26634 (10538)	804.2035	5.88	PA(44:6)
0.0496	17061	1.00 (0.00)	17061 (21850)	987.2195	8.29	PC(52:4)
0.0493	0.0000289	34599 (38083)	1.00 (0.00)	739.5423	7.09	PC(P-34:3)
0.00991	1.6788	9949 (19896)	16702 (4294)	677.5172	7.5	PE(31:0)
0.0495	12368	1.00 (0.00)	12368 (14743)	895.7634	9.89	PE(48:1)
0.0053	0.0000245	40783 (29492)	1.00 (0.00)	751.5478	7.51	PE(O-38:5)
0.0496	11351	1.00 (0.00)	11351 (14087)	762.545	8.88	PE(P-40:2)
0.00529	0.0001619	6177 (4234)	1.00 (0.00)	895.5632	7.77	PS(44:4)
0.0497	0.0000629	15897 (20229)	1.00 (0.00)	786.6563	8.11	SM(d40:1)
0.0494	4330.3	1.00 (0.00)	4330 (4875)	919.2997	9.43	TG(56:0)
0.0103	12072	1.00 (0.00)	12072 (11414)	914.8049	10.09	TG(56:2)
0.0498	11131	1.00 (0.00)	11131 (13860)	931.8561	10.55	TG(58:6)
0.0495	7027.2	1.00 (0.00)	7027 (8339)	1330.2275	10.68	TG(60:6)
0.0495	5873.2	1.00 (0.00)	5873 (6747)	994.3225	9.6	TG(62:4)
0.0102	13767	1.00 (0.00)	13767 (11703)	990.9064	10.39	TG(62:6)
0.006	0.00000441	226910 (277890)	1.00 (0.00)	2422.5576	10.06	Unknown
0.0053	0.00015721	6361 (4490)	1.00 (0.00)	230.0961	0.84	Unknown
0.0056	0.0000355	28193 (27142)	1.00 (0.00)	2466.6545	10.04	Unknown
0.0101	0.00018729	5339 (4204)	1.00 (0.00)	1051.743	0.91	Unknown
0.0102	0.0000609	16423 (13694)	1.00 (0.00)	1757.2122	0.9	Unknown
0.0102	0.0001324	7553 (6332)	1.00 (0.00)	616.4398	0.9	Unknown
0.0192	4.1139	6259 (15329)	25749 (13106)	1218.3748	9.98	Unknown
0.0332	0.30033	32401 (2637)	9731 (15111)	2666.6873	10.87	Unknown
0.0353	22840	1.00 (0.00)	22840 (17718)	2665.6724	10.87	Unknown
0.0353	46351	1.00 (0.00)	46351 (36025)	1963.527	9.51	Unknown

0.0356	177150	1.00 (0.00)	177153 (164451)	2398.612	10	Unknown
0.0377	0.36611	21463 (4728)	7858 (12183)	720.7758	7.33	Unknown
0.00397	17689	1.00 (0.00)	17689 (9488)	1156.3372	8.71	Unknown
0.00414	0.17838	34272 (3435)	6114 (14973)	1371.3169	9.3	Unknown
0.0453	3.1572	26471 (32099)	83575 (22538)	742.0622	6.96	Unknown
0.0461	0.46808	43864 (9499)	20532 (24620)	1450.3577	10.01	Unknown
0.0493	0.0000298	33532 (36788)	1.00 (0.00)	613.4736	0.91	Unknown
0.0493	0.0000746	13396 (14707)	1.00 (0.00)	1707.4868	9.83	Unknown
0.0493	0.0000778	12852 (14208)	1.00 (0.00)	1486.4329	9.52	Unknown
0.0493	2465.2	1.00 (0.00)	2465 (2715)	879.1064	0.9	Unknown
0.0493	4264.5	1.00 (0.00)	4264 (4679)	1119.3195	7.75	Unknown
0.0494	0.0000951	10518 (11765)	1.00 (0.00)	896.8426	10.38	Unknown
0.0494	4715.8	1.00 (0.00)	4716 (5287)	1157.9312	7.66	Unknown
0.0494	4817.5	1.00 (0.00)	4818 (5546)	1521.3494	10.29	Unknown
0.0494	5011	1.00 (0.00)	5011 (5749)	2134.552	10.21	Unknown
0.0494	8722.2	1.00 (0.00)	8722 (9979)	1509.02	0.9	Unknown
0.0494	10509	1.00 (0.00)	10509 (11699)	819.5858	0.93	Unknown
0.0494	19412	1.00 (0.00)	19412 (22111)	1039.321	8.48	Unknown
0.0494	21134	1.00 (0.00)	21134 (24081)	1021.2103	7.42	Unknown
0.0494	0.00022753	4395 (4852)	1.00 (0.00)	1489.4008	9.52	Unknown
0.0495	3046.8	1.00 (0.00)	3047 (3624)	942.8744	7.2	Unknown
0.0495	0.00015042	6648 (7733)	1.00 (0.00)	1888.3723	6.97	Unknown
0.0495	31044	1.00 (0.00)	31044 (37135)	405.2786	1.03	Unknown
0.0497	8050	1.00 (0.00)	8050 (10315)	1053.9314	10.24	Unknown
0.0498	15306	1.00 (0.00)	15306 (20090)	1325.2697	10.68	Unknown
0.00528	0.0000292	34290 (22915)	1.00 (0.00)	2578.6462	10.92	Unknown
0.00528	0.0000386	25884 (17526)	1.00 (0.00)	2591.6511	10.85	Unknown
0.00528	0.0000403	24786 (16535)	1.00 (0.00)	2115.4683	10.16	Unknown
0.00528	0.00010753	9300 (6250)	1.00 (0.00)	620.6424	10.5	Unknown
0.00534	0.0000444	22520 (18122)	1.00 (0.00)	1017.2298	7.27	Unknown
0.00536	0.0000104	96483 (80024)	1.00 (0.00)	2105.546	10.18	Unknown

CE: cholesterol ester; Cer: ceramide; CL: cardiolipin; LacCer: lactosylceramide; LPC: lysophosphatidylcoline; LPE: lysophosphatidyletanolamine; MG: monoacylglycerol; PA: phosphatidic acid; PC: phosphatidylcholine; PE: phosphatidyletanolamine; PS: phosphatidylserine; SM: sphingomyelin; TG: triacylglycerol.

Table S2. Differentially expressed genes in HeLa cells transfected with a plasmid expressing SrfJ compared to HeLa cells transfected with an empty vector

Gene symbol	Description	*p* value	Fold change (linear)
TNFRSF9	Tumor necrosis factor receptor superfamily, member 9	0.0025	-5.76
CREB5	Camp responsive element binding protein 5	0.0006	-5.3
IFIT2	Interferon-induced protein with tetratricopeptide repeats 2	1.19E-05	-5.26
EGR1	Early growth response 1	0.0313	-3.51
IL6	Interleukin 6	0.0022	-3.21
IFIH1	Interferon induced, with helicase C domain 1	3.21E-05	-2.84
CLGN	Calmegin	3.92E-05	-2.64
CCL5	Chemokine (C-C motif) ligand 5	0.0006	-2.61
TNFAIP3	Tumor necrosis factor, alpha-induced protein 3	0.0042	-2.49
CYR61	Cysteine-rich, angiogenic inducer, 61	0.0044	-2.47
CHAC1	Chac glutathione-specific gamma-glutamylcyclotransferase 1	0.0017	-2.34
RELB	V-rel avian reticuloendotheliosis viral oncogene homolog B	8.55E-05	-2.33
CXCL8	Chemokine (C-X-C motif) ligand 8	0.0013	-2.33
ABCC9	Transcript identified by aceview, Entrez Gene ID(s) 10060	0.0004	-2.32
CYP39A1	Cytochrome P450, family 39, subfamily A, polypeptide 1	0.0160	-2.28
CLDN1	Claudin 1	0.0052	-2.26
MAP2K1	Transcript identified by aceview, Entrez Gene ID(s) 5604	0.0472	-2.25
TMEM200A	Transmembrane protein 200A	0.0021	-2.18
F3	Coagulation factor III (thromboplastin, tissue factor)	0.0123	-2.18
ANKRD6	Ankyrin repeat domain 6	0.0036	-2.15
KLF4	Kruppel-like factor 4 (gut)	0.0035	-2.11
OR51B4	Olfactory receptor, family 51, subfamily B, member 4	0.0010	-2.07
NOCT	Nocturnin	0.0007	-2.07
CXCL11	Chemokine (C-X-C motif) ligand 11	0.0014	-2.05
BACH2	Memczak2013 ALT_ACCEPTOR, ALT_DONOR, coding, INTERNAL, intronic best transcript NM_001170794	0.0035	-2.05
DDIT3	DNA-damage-inducible transcript 3	0.0008	-2.04
OR4K5	Olfactory receptor, family 4, subfamily K, member 5	0.0196	-2.03

GBP5	Guanylate binding protein 5	0.0083	-2.02
OIT3	Oncoprotein induced transcript 3	0.0002	-2.02
MMP19	Matrix metallopeptidase 19	0.0039	-2.02
L3MBTL1	L(3)mbt-like 1 (Drosophila)	0.0101	-2
PLD1	Phospholipase D1, phosphatidylcholine-specific	0.0025	2.06
ZKSCAN4	Zinc finger with KRAB and SCAN domains 4	0.0022	2.07
RAB26	RAB26, member RAS oncogene family	0.0100	2.08
VPS35	Retromer complex component	0.0069	2.12
PDK4	Pyruvate dehydrogenase kinase, isozyme 4	0.0057	2.13
KPNA7	Karyopherin alpha 7 (importin alpha 8)	0.0116	2.18
HPGD	Hydroxyprostaglandin dehydrogenase 15-(NAD)	0.0024	2.22
TUBGCP3	Memczak2013 ANTISENSE, coding, INTERNAL, intronic best transcript NM_006322	0.0129	2.24
TTLL1	Tubulin tyrosine ligase-like family member 1	0.0086	2.25
METTL7A	Methyltransferase like 7A	0.0296	2.25
VIT	Vitrin	0.0126	2.49
ABCA1	ATP binding cassette subfamily A member 1	0.0010	2.56
ELF3	E74-like factor 3 (ets domain transcription factor, epithelial-specific)	0.0017	2.8
CD180	CD180 molecule	0.0054	2.99

Table S3. Differentially expressed genes in RAW264.7 cells infected with wild-type *S. enterica* serovar Typhimurium compared to cells infected with a *srfJ* mutant

Gene symbol	Description	*p* value	Fold change (linear)
IL1A	Interleukin 1 alpha	2.95E-05	-3.02
XAF1	XIAP associated factor 1	1.50E-05	-2.92
IL1B	Interleukin 1 beta	8.81E-05	-2.65
CD46	CD46 antigen, complement regulatory protein	0.0017	-2.61
SERPINB2	Serine (or cysteine) peptidase inhibitor, clade B. member 2	5.50E-05	-2.56
UPP1	Uridine phosphorylase 1	0.0004	-2.51
IRF7	Interferon regulatory factor 7	0.0010	-2.48
TM4SF1	Transmembrane 4 superfamily member 1	0.0017	-2.46
OLFR391-PS	Olfactory receptor 391, pseudogene	0.0028	-2.43
USP18	Ubiquitin specific peptidase 18	0.0273	-2.38
ISG20	Interferon-stimulated protein	0.0005	-2.35
EFCAB8	EF-hand calcium binding domain 8	0.0421	-2.33
TMED11	Transmembrane emp24 protein transport domain containing	0.0265	-2.26
GBP2	Guanylate binding protein 2	0.0003	-2.26
LRRC18	Leucine rich repeat containing 18	0.0015	-2.25
OLFR1082	Olfactory receptor 1082	0.0165	-2.25
OLFR902	Olfactory receptor 902	0.0445	-2.2
TCF7L2	Transcription factor 7 like 2, T cell specific, HMG box	0.0016	-2.19
GSTP2	Glutathione S-transferase, pi 2	0.0099	-2.16
FAM107B	Family with sequence similarity 107, member B	0.0101	-2.16
GPR55	G protein-coupled receptor 55	0.0018	-2.16
SLY	Sycp3 like Y-linked	0.0347	-2.15
RMDN2	Regulator of microtubule dynamics 2	0.0065	-2.15
CCL5	Chemokine (C-C motif) ligand 5	0.0026	-2.15
CCRL2	Chemokine (C-C motif) receptor-like 2	0.0069	-2.13
MMP8	Matrix metallopeptidase 8	9.71E-05	-2.12
GLT1D1	Glycosyltransferase 1 domain containing 1	0.0413	-2.11
LACC1	Laccase (multicopper oxidoreductase) domain containing 1	0.0442	-2.1
IL12B	Interleukin 12b	0.0268	-2.1
SULT1E1	Sulfotransferase family 1E, member 1	0.0015	-2.1

ZFP936	Zinc finger protein 936	0.0298	-2.09
U90926	Cdna sequence U90926	0.0003	-2.09
CD69	CD69 antigen	0.0076	-2.05
CBLN1	Cerebellin 1 precursor protein	0.0006	-2.05
LRRC16A	Leucine rich repeat containing 16A	0.0071	-2.03
CLEC2D	C-type lectin domain family 2, member d	0.0280	-2.02
MARCO	Macrophage receptor with collagenous structure	0.0010	-2
ABCC2	ATP-binding cassette, sub-family C (CFTR/MRP), member 2	0.0026	2.01
PCYT1B	Phosphate cytidylyltransferase 1, choline, beta isoform	0.0262	2.03
NHLH2	Nescient helix loop helix 2	0.0405	2.04
PRLH	Prolactin releasing hormone	0.0027	2.04
TSPAN6	Tetraspanin 6	0.0095	2.04
CCDC154	Coiled-coil domain containing 154	0.0113	2.04
SERPINA1A	Serine (or cysteine) peptidase inhibitor, clade A, member 1A	0.0120	2.04
OLFR1480	Olfactory receptor 1480	0.0259	2.06
GAL3ST2	Galactose-3-O-sulfotransferase 2	0.0058	2.07
SULT3A1	Sulfotransferase family 3A, member 1	0.0129	2.1
GIMAP3	Gtpase, IMAP family member 3	0.0084	2.11
TTLL11	Tubulin tyrosine ligase-like family, member 11	0.0159	2.11
OLFR259	Olfactory receptor 259	0.0065	2.11
OLFR1026	Olfactory receptor 1026	0.0490	2.13
ATAD5	Atpase family, AAA domain containing 5	0.0316	2.14
SPIN2E	Spindlin family, member 2E	0.0125	2.14
KPTN	Kaptin	0.0480	2.16
OLFR199	Olfactory receptor 199	0.0032	2.18
CNBD1	Cyclic nucleotide binding domain containing 1	0.0339	2.18
OLFR924	Olfactory receptor 924	0.0073	2.19
CHRDL1	Chordin-like 1	0.0041	2.26
B3GALT1	UDP-Gal:betaglcnac beta 1,3-galactosyltransferase, polypeptide 1	0.0317	2.27
VCAN	Versican	0.0299	2.29
VMN2R17	Vomeronasal 2, receptor 17	0.0022	2.31
ATP2C2	ATPase, Ca++ transporting, type 2C, member 2	0.0029	2.36

RBBP9	Retinoblastoma binding protein 9	0.0032	2.37
DUSP21	Dual specificity phosphatase 21	0.0292	2.44
VMN1R54	Vomeronasal 1 receptor 54	0.0118	2.53
LCE1C	Late cornified envelope 1C	0.0284	2.58
IPW; SNORD116	Imprinted gene in the Prader-Willi syndrome region; small nucleolar RNA, C/D box 116 cluster	0.0002	2.97
MUP19	Major urinary protein 19	0.0372	3.05

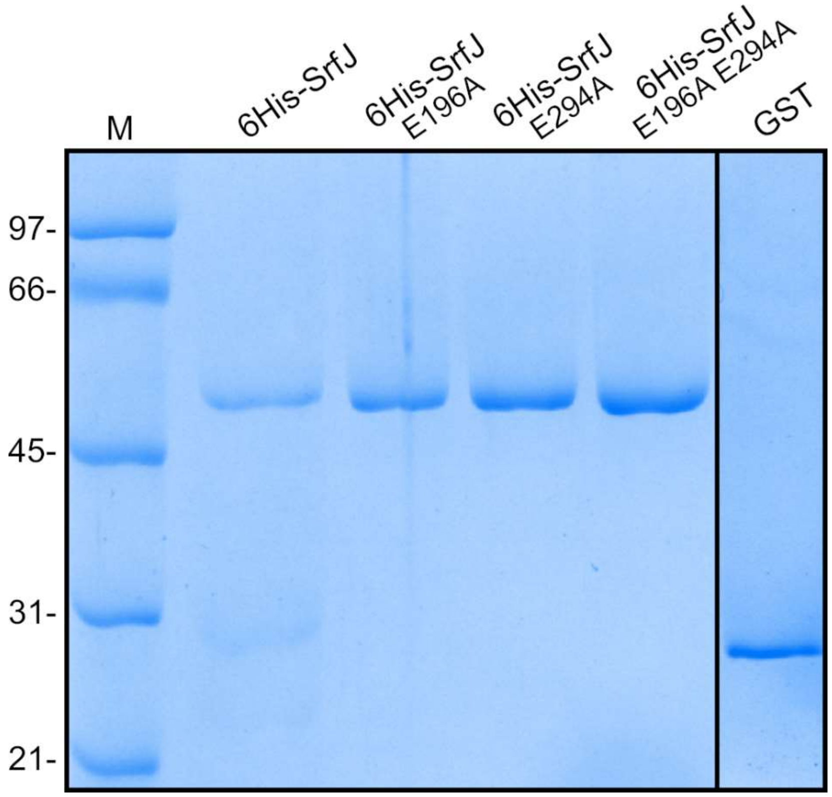

Figure S1. Purified proteins used in the experiment shown in Figure 1. SDS-polyacrylamide gel electrophoresis analysis of purified GST, 6His-SrfJ and derived mutated forms of this protein. The gel was stained with Coomassie blue. M, molecular weight markers in kDa.
